# Systematic review update and meta-analysis of randomized and non-randomized controlled trials of ovarian stimulation versus artificial cycle for endometrial preparation prior to frozen embryo transfer in women with polycystic ovary syndrome

**DOI:** 10.1186/s12958-022-00931-4

**Published:** 2022-04-02

**Authors:** Yingying Zhang, Ling Wu, Tin Chiu Li, Chi Chiu Wang, Tao Zhang, Jacqueline Pui Wah Chung

**Affiliations:** 1 Department of Obstetrics and Gynaecology, The Chinese University of Hong Kong, Hong Kong SAR, China; 2 Department of Obstetrics and Gynaecology, Li Ka Shing Institute of Health Sciences, School of Biomedical Sciences, Chinese University of Hong Kong -Sichuan University Joint Laboratory in Reproductive Medicine, The Chinese University of Hong Kong, Hong Kong SAR, China

**Keywords:** Endometrial preparation, HRT, Stimulated, PCOS, Frozen embryo transfer

## Abstract

**Purpose:**

This systematic review and meta-analysis aimed to compare the short-term reproductive and long-term obstetric outcomes after endometrial preparations by ovarian stimulation protocols and hormone replacement therapy (HRT) in women with polycystic ovary syndrome (PCOS) prior to frozen embryo transfer (FET).

**Method:**

PubMed, EMBASE, Web of Science and the Cochrane Library were searched to identify relevant studies. Primary outcome was live birth rate, secondary outcomes included the rates of clinical pregnancy, miscarriage, implantation and hCG-postive, cycle cancellation, ectopic pregnancy, preterm birth, preeclampsia, gestational hypertension, gestational diabetes mellitus and abnormal placentation.

**Results:**

Nine studies, including 8327 patients with PCOS, were identified. Live birth rate was significantly higher (RR = 1.11, 95% CI = 1.03–1.19) and miscarriage rate (RR = 0.60, 95% CI = 0.46–0.78) was significantly lower in stimulated protocol compared to the rates in HRT. While the rates of ongoing pregnancy, clinical pregnancy, implantation, hCG-positive, cycle cancellation and ectopic pregnancy showed no significant difference between the two protocols. Compared HRT with different stimulation protocols, significantly higher clinical pregnancy rate (RR = 1.54, 95% CI = 1.20–1.98) were found in letrozole group, but not in the other subgroups. For the obstetric outcomes, the preterm birth and preeclampsia rates were significantly lower in the stimulated group compared to that in the HRT group (RR = 0.85, 95% CI = 0.74–0.98; RR = 0.57, 95% CI = 0.40–0.82, respectively), while gestational hypertension, gestational diabetes mellitus and abnormal placentation rates showed no significant difference.

**Conclusions:**

The present data suggest that ovarian stimulation protocol as an endometrial preparation regimen prior to FET might be superior to HRT protocol with a significantly higher rate of live birth, lower risk of miscarriage, preterm birth and preeclampsia. Our study showed stimulated protocol is better than HRT regimen as an endometrial preparation for women with PCOS. However, quality of the evidence is low, more well-designed RCT studies are still needed to confirm the results before clinical recommendation, particularly direct comparisons between letrozole and other stimulated regimens.

**Supplementary Information:**

The online version contains supplementary material available at 10.1186/s12958-022-00931-4.

## Background

Women with polycystic ovary syndrome (PCOS) are considered patients with impaired reproductive ability regardless of ovulatory state [1]. The clinical features related to PCOS may lead to endometrial dysfunction in women with PCOS, including impairment in the expression of sex hormone receptors, insulin resistance, and glucose transport in endometrium [2]. Considering the prevalence of insulin resistance in women with PCOS, metformin was proposed as one of the treatments for PCOS [3]. In addition, in-vitro fertilization embryo-transfer (IVF-ET) has proven to be a beneficial treatment option for women with PCOS. However, they are more likely to result in a significantly higher occurrence of iatrogenic ovarian hyperstimulation syndrome (OHSS) in about 7 folds than women without PCOS [4]. The early-onset of OHSS is related to exaggerated ovarian response to gonadotrophin stimulation and administration of hCG, while the late-onset is related to the endogenous hCG from trophoblast where conception occurs [5]. To prevent OHSS, freezing all embryos and waiting for a suitable time for embryo transfer are proposed [6]. Indeed, a randomized clinical trial with a large sample size showed that women with PCOS who underwent frozen-embryo transfer (FET) had a lower incidence of OHSS (1.3% vs. 7.1%) and pregnancy loss (22% vs. 32.7%) and a higher rate of live birth (49.3% vs. 42%), compared with women with PCOS underwent fresh embryo transfer cycles [7]. To achieve a better pregnancy outcome, selective FET is considered a cost-effective treatment for those infertile women with PCOS in many IVF centers [6, 8].

Synchronization between receptive endometrium and embryo development is critical for pregnancy establishment [9]. Natural protocol is not commonly used since women with PCOS usually have irregular menstruation or oligo-anovulation [10], but artificial cycle and ovarian stimulation have been wildly applied. Hormone replacement therapy (HRT) is used in women with PCOS in an artificial cycle where the endometrium is prepared with programmed estrogen and progesterone, leading to a fixed time for ET. In addition, the application of GnRH agonist/antagonist in HRT cycle is considered as a strategy to achieve pituitary down-regulation and avoid spontaneous ovulation, thus preventing cycle cancellation. Stimulation protocols use ovulation stimulation medications, including clomiphene citrate (CC), letrozole, and gonadotropins to mimic the natural process of follicular development and facilitate endogenous estradiol through ovulation induction, which is different from that by exogenous estradiol in HRT protocols. hCG is commonly employed as a surrogate LH surge to stimulate the maturation of oocyte and increase the endometrial receptivity. In addition, the reported pregnancy outcomes for women with PCOS were different among varied strategies of stimulation drugs [11] and which stimulation drug is superior has yet determined.

A recent meta-analysis including 4 retrospective studies with 2933 women with PCOS published from 2013 to 2019, suggested that the pregnancy outcomes, including rates of live birth, ongoing pregnancy, clinical pregnancy, implantation and miscarriage rate were not significantly different between artificial and ovarian stimulation cycles [12]. After that, 1 RCT study [13] and 4 retrospective studies [14–17] on the topic have been published, suggesting the therapeutic effects might be different among various ovulation stimulation regimens. Which stimulation regimen superior to HRT has yet been analyzed in the previous review paper. In addition, obstetric outcomes have been reported to be associated with different types of endometrial preparations [18], but they were not evaluated before probably because of the limited studies in PCOS.

This study is a systematic review update, meta-analysis and sub-group analysis including more studies, aiming to compare the differences in short-term reproductive and long-term obstetric outcomes between artificial and stimulation protocols prior to FET in women with PCOS.

## Materials and methods

### Search strategy

Literatures were systematically searched from PubMed, EMBASE, Web of Science and The Cochrane Library for publications in English unitil May 27^th^, 2021, for studies reporting on comparison of clinical outcomes between artificial and stimulating protocols in women with PCOS undergoing FET. The search strategy used the following combined search terms: (((((embryo[Title/Abstract]) OR (blastocyst[Title/Abstract])) AND (transfer*[Title/Abstract])) OR (Embryo Transfer[Mesh])) AND ((((("Cryopreserv*"[Title/Abstract]) OR ("freez*"[Title/Abstract])) OR ("vitrifi*"[Title/Abstract])) OR ("frozen"[Title/Abstract])) OR ("frozen-thawed"[Title/Abstract]))) AND (("polycystic ovary syndrome"[Title/Abstract]) OR ("Polycystic Ovary Syndrome"[Mesh])). Meanwhile, references cited in the original literatures were also searched. The registrated PROSPERO number was CRD42021258267.

### Inclusion and exclusion criteria

Inclusion criteria were as follows: (1) women with diagnosed PCOS undergoing FET; (2) comparative studies including randomized controlled trials (RCT), cohort studies and case–control studies; (3) report of clinical results including reproductive and obstetrical outcomes; (4) comparison of endometrial preparation methods between artificial and stimulation protocols. Exclusion criteria were as follows: (1) studies not relevant to PCOS, FET or endometrial preparation; (2) studies not compared between artificial and stimulating protocols; (3) case report, review, letters, meta-analysis, comments, editorial, protocols and animal experiments and (4) studies without available data for analysis.

### Study selection

All the identified studies were screened by two independent reviewers (YYZ and LW). Data extraction and assessment of study quality for the included studies were performed by the two reviewers independently and disagreements were resolved by an independent external reviewer (TZ).

### Data extraction and quality assessment

All evidence extracted from the studies were symmaized and described qualitatively in Table 1. The essential characteristics of studies were presented, including the first author's name, publication year, study design, diagnosis criteria of PCOS, age, BMI, method of endometrial preparation, the quality of embryos transferred, reproductive and obstetric outcomes. The Cochrane Risk of Bias tool evaluated the quality of the RCT study [19]. Five domains were included in the assessment, including random sequence generation, allocation concealment, blinding of participants and personnel, incomplete outcome data, selective reporting and other sources of bias. Newcastle–Ottawa Scale (NOS) was applied for quality assessment in cohort studies [20]. NOS assesses the risk of selection bias, the comparability of the groups, ascertainment of exposure, and assessment of outcomes of included studies. A higher number of stars represents a higher study quality. Study with > 7 scores were considered high-quality overall study 5–7 scores as moderate-quality and < 5 scores as low-quality.

**Table 1:** Basic characteristics of included studies

Author year	Study period	Study design	PCOS diagnostic criteria	Participants	Age, year	BMI, kg/m^2^	HRT protocols	Stimulated protocols	Number of embryo transferred	Time of embryo transferred
Li Li 2021	2017–2020	retrospective	2003 Rotter- dam criteria	1234/200	HRT 29.6 ± 3.1; rFSH 29.7 ± 2.9	HRT 23.7 ± 3.9; rFSH 23.7 ± 3.9	Estradiol valerate 4 mg QD from day 2/3 for 5 days followed by estradiol valerate 6 mg QD 6–8; vaginal supplementation with progesterone 90 mg daily were added days when the endometrial thickness reached 7 mm and the serum progesterone level was below 1.5 ng/mL	Gonarfen (37.5–75 IU) QD from day 5; triggered by hCG (5000 IU) or recombinant hCG (250 μg)	1 or over	Cleavage stage embryos
Jie Zhang 2021	2010–2018	retrospective	2003 Rotter- dam criteria	1168/1259	NA	NA	Estradiol from day3; when the endometrial thickness was 7 mm, exogenous progesterone supplementation was provided	Letrozole, 5 mg QD from day 3 to day 7; in case of a dominant follicle < 14 mm on day 10, a low dosage of hMG was supplemented with incremental doses of 37.5 IU if needed; triggered by hCG	1 or over	Cleavage stage embryos/blastocysts
Nardin Aslih 2021	2016–2018	retrospective	NA	80/25	HRT 33.4 ± 5.3; letrozole 35.2 ± 5.3	HRT 28.0 ± 5.6; letrozole 30.0 ± 7.4	Estradiol 2 mg TID from day 3 for at least 8 days; on day 8 progesterone (oral dydrogesterone 10 mg TID or vaginal micronized progesterone 100 mg TID or micronized progesterone gel 90 mg BID) was started when endometrial thickness was more than 8 mm	Letrozole, 2.5 mg BID from day 5 to day 9; triggered by 250 mcg recombinant hCG	1	Cleavage stage embryos/blastocysts
Azadeh Hosseini-Najarkolaei 2020	2018–2020	ART	2003 Rotter- dam criteria	59/57	HRT 29.45 ± 0.42 vs. 30.12 ± 0.33	HRT 26.10 ± 0.48; letrozole + hMG 25.70 ± 0.46	Estradiol valerate 4 mg QD for 3 days and then 6 mg QD; on day 7 of estradiol administration, progesterone 50 mg QD IM for 2 or 3 days	Letrozole, 2.5 mg BID from day 3 to day 7 and hMG 75–150 nIU QD from day5 to day9; triggered by 1000 IU hCG	1 or 2	Cleavage stage embryos
Yuanyuan Man 2020	2014–2017	retrospective	2003 Rotter- dam criteria	1224/88	HRT 28 (26–31); HMG 28 (26–30)	HRT 24.43 (21.81–27.31); HMG 24.66 (21.38–28.73)	Estradiol valerate 4 mg QD from day 2/3 for 5 days followed by estradiol valerate 6 mg QD for 5 days; 5 days before blastocyst transfer oral dydrogesterone 30 mg QD was started	hMG 37.5/75 IU QD from day3/5; triggered by 4000–8000 IU hCG	1 or 2	Blastocysts
Jie Zhang 2019	2011–2016	retrospective	2003 Rotter- dam criteria	850/1236	HRT 29.07 ± 3.32 vs. 28.95 ± 3.27	HRT 23.34 ± 3.68; letrozole + hMG 23.11 ± 3.62	oral 17b-E2 2 mg TID from day2/3; when endometrium thickness over 7 mm, vaginal progesterone 400 mg QD were taken	Letrozole, 5 mg QD from day 3 to day 7; in case of a dominant follicle < 14 mm on day 10, a daily dosage of 75 IU hMG was supplemented with incremental doses of 37.5 IU if needed; triggered by 5000 IU hCG	1 or 2	Cleavage stage embryos/blastocysts
Lee KH 2017	2014–2015	retrospective	NA	119/45	NA	NA	6 mg estradiol valerate from day3 + 50 mg; progesterone, IM	Letrozole, 5 mg QD from day 3 to day 7	NA	Cleavage stage embryos
Jian Mei Yu 2015	2010–2012	retrospective	2003 Rotter- dam criteria	273/262	HRT 30.51 ± 3.35 vs. 30.48 ± 3.34	HRT 22.74 ± 2.09; hMG 22.58 ± 2.17	Estradiol valerate 2 mg BID from day3; progesterone 40 mg IM when endometrium thickness reached at 8 mm	hMG 75 IU QD from day 5 to day9; on day 10, the dose was increased in 75–150 IU for 5–10 days when needed; triggered by 5000–10,000 IU hCG	1 or over	Cleavage stage embryos
Yan Jun Hu 2014	2011–2012	retrospective	2003 Rotter- dam criteria	76/40/32	HRT 28.9 ± 3.0 vs. 30.1 ± 3.7 vs. 28.7 ± 4.1	HRT 22.6 ± 2.5; letrozole 22.8 ± 2.0; hMG 22.5 ± 2.3	Oestrogen 2–4 mg QD for 5–7 days followed by 4–6 mg QD for 5–7 days; progesterone 80 mg QD was commenced when the endometrium thickness reached at 8 mm	Letrozole group: letrozole, 5 mg QD from day 3 to day 7; hMG 75 IU were added if the follicular diameter was less than 14 mm on day10 with dose increments of 37.5 IU every 5–7 days when needed; triggered by 10,000 IU hCG. hMG group: hMG 75 IU QD from day 3; on day 10, the dose was increased in 37.5 IU for 5–7 days when needed; triggered by 10,000 IU hCG	1 or over	Cleavage stage embryos

*NA* not available

### Outcomes

The primary outcome was the live birth rate (LBR). The secondary outcomes were reproductive outcomes, including clinical pregnancy rate (CPR), miscarriage rate (MR), implantation rate (IR), hCG-positive rate (HPR), cycle cancellation rate (CCR); and obstetric outcomes, including ectopic pregnancy rate (EPR), preterm birth rate (PBR), preeclampsia rate (PR), gestational hypertension rate (GHR), gestational diabetes mellitus rate (GDMR) and abnormal placentation rate (APR).

### Statistical analysis

Review Manager 5 (RevMan Version 5.4 Software, Copenhagen, Denmark) was used to perform the meta-analysis. Risk ratio (RR) and 95% confidence interval (CI) were used as effect sizes for calculated outcomes. Mantel–Haenszel was applied in statistical analysis of the outcome data from included studies and random effects models were used. Heterogeneity was assessed by the I^2^ and P_Q_ from Cochran's Q test and publication bias for the outcomes in the total analysis was evaluated in funnel plots [21]. Besides, fixed effects models were used for sensitivity analysis, in which meta-analysis was repeatedly conducted to assess the influence of the most extreme study with the exclusion of this study. A *p*-value less than 0.05 was considered to be statistically significant. Sub-group analyses were conducted to compare the effect of HRT with different simulated regimes, including letrozole alone, HMG alone, letrozole followed by HMG (letrozole + HMG), letrozole followed by HMG or not (letrozole ± HMG) and rFSH alone. In addition, comparison between different study type (RCT verses non-RCT) and stage of embryo transferred (cleavage-stage embryo verses blastocyst verses mix of cleavage and blastocyst) were conducted in subgroup analysis. Due to the incomplete data about the use of GnRH agonist/antagonist for avoiding ovulation in patients with received HRT, hCG for triggering ovulation and luteal phase support, subgroup analyses were not conducted for those factors.

## Results

### Study selection

**Fig. 1:**
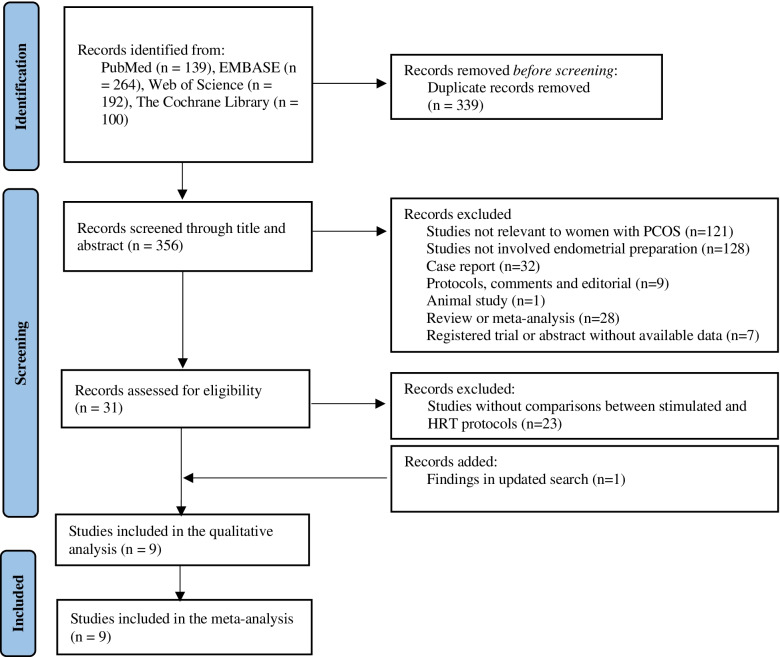
Prisma flowchart of study selection

Six hundred ninety-six potential studies were yielded in screening, including 139 from PubMed, 264 from EMBASE, 194 from Web of Science and 100 from The Cochrane Library. After the removal of 339 duplicated studies and 348 non-relevant studies according to inclusion and exclusion criteria. Nine studies that compared the clinical outcomes between stimulation cycles and HRT cycles were included in this meta-analysis (Fig. 1). As shown in Table 1, 8 studies were retrospective cohort studies and 1 was an RCT. The studies were conducted all in Asia, including Iran (*n* = 1), Israel (*n* = 1), Korea (*n* = 1), and China (*n* = 6). The sample size ranged from 105 to 3131. As for the diagnosis criteria of PCOS, 7 of them applied the 2003 Rotterdam criteria and 2 did not mention about it. The mean age for the study population ranged from 28 to 35.2 years old, BMI ranged from 22.5 to 30 kg/m^2^. In addition, the regimes for the HRT were not the same among the included studies. The dosage of oral estrogen ranged from 2 to 6 mg daily; the routes and dosage of progesterone varied, including oral, vaginal supplementation, and intramuscular injection, with different dosages. Most (7/9) of the included studies transferred 1 or more embryos, 1 study transferred 1 embryo and 1 study did not mention about it. As for the stage of embryo transferred, 5 studies transferred cleavage embryo, while 1 study only transferred blastocyst embryos and 3 studies transferred both cleavage and blastocyst embryos. The overall quality assessment is presented in Table 2. One cohort study was rated as moderate quality (less than 7 scores), the rest were ranked as high quality (7 or over 7 score). The RCT study was rated as low risk in selective reporting and complete outcome data, but the unclear risk in random sequence generation, allocation concealment, blinding and other sources of bias.

**Table 2:** Quality assessment for included non-RCT studies

Author year	Li li 2021	Jie Zhang 2021	Nardin Aslih 2021	Yuanyuan Man, 2020	Jie Zhang 2019	Lee KH 2017	Jian Mei Yu 2015	Yan Jun Hu 2014
Representativeness of the exposed cohort	*	*	*	*	*	*	*	*
Selection of the non-exposed cohort	*	*	*	*	*	*	*	*
Ascertainment of exposure	*	*	*	*	*	*	*	*
Demonstration that outcome of interest was not present at start of study	/	/	/	/	/	/	/	/
Comparability of cohorts on the basis of the design or analysis	**	**	**	**	**	/	**	**
Assessment of outcome	*	*	*	*	*	*	*	*
Was follow-up long enough for outcomes to occur	*	*	*	*	*	*	*	*
Adequacy of follow-up of cohorts	*	*	*	*	*	*	*	*
Summary quality score	8	8	8	8	8	6	8	8

### Primary outcome

#### Live birth rate

**Fig. 2:**
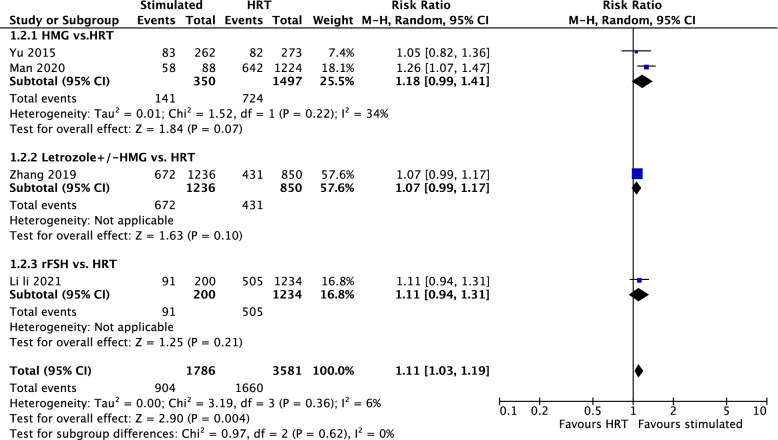
Live birth rate between the stimulated and HRT cycles

Five retrospective studies, with 1811 PCOS receiving stimulation protocol and 3661 PCOS receiving HRT reported. Significantly higher LBR (RR = 1.14; 95% CI = 1.01–1.29; I^2^ = 56%, P_Q_ = 0.06) was found in stimulation group than that in HRT group as shown in Supplementary Fig. 1. Based on sensitivity analysis, 1 study (Aslih 2021) [14] was found with publication bias (Supplementary Fig. 2A). After excluding this study, significantly higher LBR (RR = 1.11; 95% CI = 1.03–1.19; I^2^ = 6%, P_Q_ = 0.36) was still observed in the stimulation group (Fig. 2). Subgroup analyses showed HMG (RR = 1.18; 95%CI = 0.99–1.41; *P* = 0.07), letrozole ± HMG (RR = 1.07; 95% CI = 0.99–1.17; *P* = 0.10) and rFSH (RR = 1.11; 95% CI = 0.94–1.31; *P* = 0.21) had similar LBR compared with HRT. As for the stage of embryo transferred (Supplementary Fig. 3), significant difference in LBR (favouring stimulated group) between two groups was only observed in the subgroup of blastocyst (RR = 1.18; 95% CI = 1.05–1.34).

### Secondary outcomes

#### Ongoing pregnancy rate

**Fig. 3:**
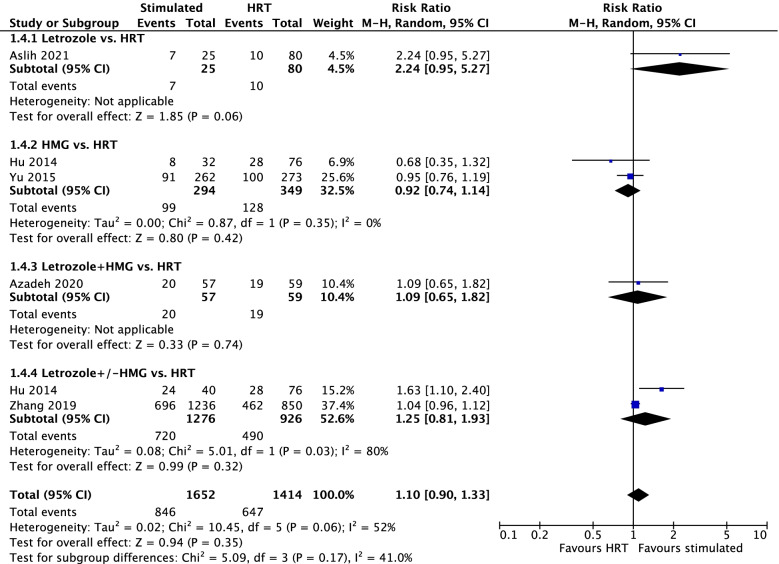
Ongoing pregnancy rate between the stimulated and HRT cycles

Six studies, which included 1697 PCOS receiving stimulation protocol and 1457 PCOS receiving HRT reported OPR. However, no significant difference (RR = 1.15; 95% CI = 0.96–1.37; I^2^ = 52%, P_Q_ = 0.07) between stimulation protocol and HRT protocol was observed (Supplementary Fig. 4). Based on sensitivity analysis (Supplementary Fig. 2B), 1 study (Lee 2017) [22] was found with publication bias. The result showed similar findings (RR = 1.04; 95% CI = 0.95–1.15; I^2^ = 7%; P_Q_ = 0.36) between the two groups after excluding this study (Supplementary Fig. 5). In addition, subgroup analyses showed letrozole (RR = 2.24; 95% CI = 0.95–5.27; *P* = 0.06), HMG (RR = 0.92; 95% CI = 0.74–1.14; *P* = 0.42), letrozole + HMG (RR = 1.09; 95% CI = 0.65–1.82; *P* = 0.74) and letrozole ± HMG (RR = 1.25; 95% CI = 0.81–1.93; *P* = 0.32) had similar OPR compared with HRT (Fig. 3). In addition, subgroup comparisons in type of study (RCT and non-RCT studies) and the stage of embryo transferred (cleavage-stage embryo, mix of cleavage-stage embryos and blastocyst) showed no statistical difference in OPR between the stimulated and HRT groups (Data not shown).

#### Clinical pregnancy rate

Eight studies including 1985 PCOS receiving stimulation protocol and 3915 PCOS receiving HRT, reported CPR. No significant difference between stimulation protocol and HRT protocol was observed between the stimulated and HRT cycles (RR = 1.09; 95% CI = 0.99–1.20; I^2^ = 47%; P_Q_ = 0.07, Supplementary Fig. 6). Subgroup analyses showed letrozole (RR = 1.54; 95% CI = 1.20–1.98; *P* < 0.001) had a significantly higher CPR than HRT, but HMG (RR = 1.09; 95% CI = 0.98–1.21; *P* = 0.13), the letrozole + HMG (RR = 1.37; 95% CI = 0.97–1.94; *P* = 0.08), letrozole ± HMG (RR = 0.98; 95% CI = 0.91–1.05; *P* = 0.53) and rFSH (RR = 1.04; 95% CI = 0.90–1.20; *P* = 0.63) had similar CPR compared to HRT (Supplementary Fig. 7). The subgroup analysis based on type of study and embryo transferred showed similar CPR between the two groups (data not shown). As for the subgroup analysis of the stage of embryo transferred, significant higher (RR = 1.13; 95% CI = 1.00–1.29) in CPR (favouring stimulated group) was only observed in the subgroup of blastocyst based on 1 study [16].

#### Miscarriage rate

**Fig. 4:**
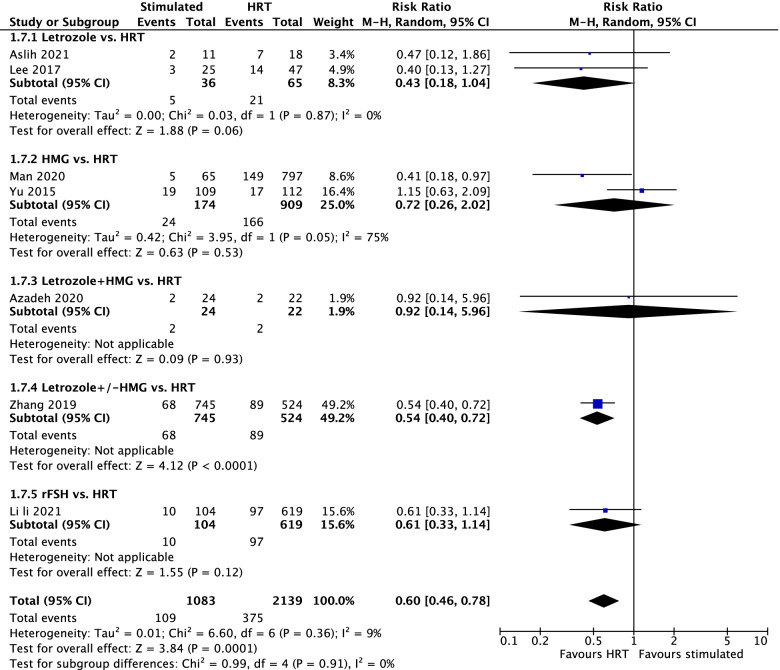
Miscarriage rate between the stimulated and HRT cycles

MR based on 1083 PCOS receiving stimulation protocol and 2139 PCOS receiving HRT in 7 studies showed significantly lower rate in the stimulated cycles (RR = 0.60; 95% CI = 0.46–0.78; I^2^ = 9%; P_Q_ = 0.36) compared with HRT cycles (Fig. 4). Subgroup analyses showed letrozole (RR = 0.43; 95% CI = 0.18–1.04; *P* = 0.06), HMG (RR = 0.72; 95% CI = 0.26–2.02; *P* = 0.53), the letrozole + HMG (RR = 0.92; 95% CI = 0.14–5.96; *P* = 0.93) and rFSH (RR = 0.61; 95% CI = 0.33–1.14; *P* = 0.12) had similar miscarriage rate to HRT, while letrozole ± HMG (RR = 0.54; 95% CI = 0.40–0.72; *P* < 0.001) had significantly lower miscarriage to HRT based on 1 study. Since there was only one RCT study, the significant difference of MR was only found in analysis based on non-RCT studies (Data not shown). As for the stage of embryo transferred, significance of MR (favouring HRT) was observed in the subgroup of blastocyst (RR = 0.41, 95% CI = 0.18–0.97) based on 1 study and in the mixed blastocyst/cleavage stage group (RR = 0.53, 95% CI = 0.40–0.71) based on 2 studies (Supplementary Fig. 8).

#### Implantation rate and hCG-positive rate

Three studies reported IR based on 3141 PCOS receiving stimulation protocol and 2423 PCOS receiving HRT and no significant difference was observed (RR = 0.99; 95% CI = 0.93–1.06; I^2^ = 0%; P_Q_ = 0.38; Supplementary Fig. 9). Subgroup analyses showed both HMG and letrozole ± HMG groups had similar IR with HRT (Supplementary Fig. 10). Six studies including 1868 PCOS receiving stimulation protocol and 3720 PCOS receiving HRT reported HPR (Supplementary Fig. 11), but no significant difference was observed (RR = 1.03; 95% CI = 0.96–1.10; I^2^ = 23%; P_Q_ = 0.26). The subgroup analyses of different stimulated drugs showed no significant difference. In addition, the subgroup analysis of study type and stage of embryo transferred for IR and HPR showed no significant difference (Data not shown).

#### Cycle cancellation rate

Four studies including 2012 PCOS receiving stimulation protocol and 2877 PCOS receiving HRT reported cycle cancellation rate and no significant difference was observed either in comparison of total stimulated cycle (RR = 0.96; 95% CI = 0.70–1.31; I^2^ = 30%; P_Q_ = 0.23) in subgroup analyses with HRT (Supplementary Fig. 12). The results demonstrated no significant difference in CCR between the stimulated and HRT groups after subgroup analysis of study types and stage of embryo transferred (Data not shown).

#### Ectopic pregnancy rate

Five studies including 313 PCOS receiving stimulation protocol and 1568 PCOS receiving HRT reported EPR and no significant difference was observed (RR = 1.55; 95% CI = 0.84–2.87; I^2^ = 0%; P_Q_ = 0.52). Subgroup analyses also showed similar EPR between HRT cycle and letrozole, HMG, letrozole + HMG and rFSH cycles (Supplementary Fig. 13). There is no significant difference in EPR between the stimulated and HRT groups after subgroup analysis of different stimulated drugs, study types and stage of embryo transferred (Data not shown).

#### Obstetric outcomes

Only 2 studies reported obstetric outcomes of preterm birth, preeclampsia, gestational hypertension, gestational diabetes mellitus and abnormal placentation (Supplementary Fig. 14). The overall RR of stimulated cycles for PBR and PR was 0.85 (95% CI = 0.74–0.98; I^2^ = 0%; P_Q_ = 0.94) and 0.57 (95% CI = 0.40–0.82; I^2^ = 0%; P_Q_ = 0.87) compared to HRT cycles. However, there were no significant difference in GHP (RR = 1.04; 95% CI = 0.47–2.27; I^2^ = 69%; P_Q_ = 0.07), GDMR (RR = 0.91; 95% CI = 0.74–1.12; I^2^ = 0%; P_Q_ = 0.65), APR (RR = 0.64; 95% CI = 0.37–1.12; I^2^ = 0%; P_Q_ = 0.93) between the stimulated and HRT cycles.

## Discussion

### Main findings

In summary, evidence from 9 studies with 8327 patients indicated that LBR was significantly higher. At the same time, MR, PBR, and PR were significantly lower in the stimulated group than the rates in the HRT group, which is different from the previous meta-analysis [12]. When the ovarian stimulation was further divided into subgroups of letrozole, HMG, letrozole + HMG, letrozole ± HMG and rFSH, CPR were significantly higher in the letrozole group but not in the other subgroups compared with HRT. This finding suggested that stimulated protocols is superior endometrial preparation for women with PCOS than artificial protocols to improve pregnancy outcomes.

### Biological plausibility of endometrial preparation in women with PCOS

The most common endometrial preparation regimen prior to FET for women with PCOS is HRT, as it is easy to manage by clinicians and more convenient for patients. The exogenous estrogen and progesterone are supposed to mimic similar hormone thyme within a regular menstrual cycle. Once pregnancy is established, exogenous estrogen and progesterone cannot be withdrawn until placenta formation due to the absence of corpus luteum. Some studies have shown that exogenous hormones might be insufficient for proper endometrial development and decidualization before and/or during pregnancy [23]. Moreover, an accumulating body of evidence showed that the pregnancy complications, such as hypertension disorder, and perinatal outcomes like large for gestational age, were significantly higher in the HRT cycles in the general population [24–26]. Thus, the current perspective prefers that the natural cycles are superior to HRT based on available low-quality evidence [27]. This evidence gives rise to whether the stimulated cycle is superior to the HRT cycle in the condition that the natural cycle does not apply to most women with PCOS.

### Reproductive outcomes

The reproductive outcomes between artificial and stimulated protocols in women with PCOS have been analyzed by a previous meta-analysis which included four retrospective studies with 2933 transfer cycles [12]. This study found the LBR was higher and MR was lower in stimulated protocols than the HRT cycles rates, but the difference was not statistically significant. Our meta-analysis recruited another five studies. The results showed significantly higher LBR and lowered MR in stimulated protocols, indicating that stimulated protocol might be superior to the artificial protocol as an endometrial preparation method before EFT for women with PCOS. The potential reason causing poorer reproductive outcomes in the HRT cycle might be associated with a lower serum progesterone level. Emerging studies have found a potentially negative association between low progesterone and LBR in women with artificial cycles [28, 29]. A recently published study showed that nearly one-third of patients receiving artificial protocol with estradiol valerate and micronized vaginal progesterone had inadequate serum progesterone (< 8.8 ng/ml ET day), and those patients were found to have significantly higher MR, lower OPR and LBR [30]. The same study group further demonstrated that individualized luteal support for women with lower serum progesterone in artificial cycle could significantly improve LBR [31]. Therefore, we assumed that the decreased serum progesterone might be one of the contributors to the unfavorable pregnancy outcomes in women with artificial cycles.

In subgroup analyses, we found that the CPR were significantly higher in the letrozole group than the HRT group, but not in the comparison between the other stimulated cycles and the HRT group. Anovulatory infertile people with PCOS might benefit from letrozole treatment [32]. The administration of letrozole could induce an acute hypoestrogenic state, which relieves the hypothalamic-pituitary axis from estrogenic negative feedback, increasing FSH production and ovarian follicle growth (Supplementary Fig. 15). Among all the recruited studies. Zhang’s study [33] has the largest sample size (*n* = 2086), accounting for 40.25% of the total sample size in the analysis of LBR. As shown in the study, letrozole group had a higher LBR (RR = 1.16, 95% CI = 0.97–1.38) and lower MR (RR = 0.49, 95% CI = 0.35–0.69) than the artificial group. In our meta-analysis, LBR was still significantly higher (RR = 1.11, 95% CI = 1.03–1.19) in the stimulated group than HRT group without inclusion of this study. Along with this study, the outcome of our meta-analysis based on a larger sample size further indicates the superiority of letrozole as the endometrial preparation compared to HRT.

However, only 1–3 studies were recruited in the subgroup of stimulation cycles. Thus, the results for the subgroup analyses might not be very robust. Nevertheless, the role of letrozole in improving endometrial receptivity has been extensively reported [34–37]. The benefit of letrozole in our subgroup analysis has also been found in the general infertile population by a large cohort study that recruited 110,722 single FET cycles and showed that the application of letrozole could improve the CPR and LBR and decrease MR compared to HRT cycles [38]. Still, the proportion of PCOS in the study population was not available [38]. The exact underlying mechanism of how letrozole enhances pregnancy outcome has not been completely elucidated yet, but it might be correlated with improved endometrial receptivity. It has been demonstrated that the reduced estrogen level during the early follicular phase induced by letrozole could lead to higher expression of estrogen receptors which could improve the endometrial receptibility [39, 40]. Besides, the application of letrozole could increase the expressions of uterine receptivity markers, including integrin, L-selectin, leukemia inhibitory factor and formation of pinopod compared to the natural cycle [41, 42].

On the other hand, the superiority of letrozole to HMG in promoting follicle development has been indicated in previous studies. A cohort study with 5901 FET cycles showed that women receiving letrozole or letrozole + HMG stimulation exhibited significantly higher CPR but lower MR than women receiving only HMG stimulation in ovulatory infertile women. At the same time, there was no significant difference in MR between letrozole and letrozole + HMG groups [43]. In our study, the letrozole group rather than the letrozole + HMG group showed significantly higher CPR than HRT cycles. Based on the potential benefits of letrozole in the literature, more studies are warranted to investigate the efficiency of different stimulated protocols in endometrium preparation, especially for women with PCOS.

### Obstetrics outcomes

The available data for analysis in obstetric outcomes between the stimulated and HRT cycles in women with PCOS undergoing FET is limited. Only two studies with 3127 patients compared HMG to HRT, letrozole ± HMG to HRT, respectively [16, 44]. The results showed that the risk of preterm birth and preeclampsia in the stimulated cycle was much lower when compared to that in the HRT cycle.

The exact mechanisms of higher risks of preterm birth and preeclampsia in women with the HRT cycle are unclear, but they might be correlated with the establishment of the corpus luteum. The formation of the corpus luteum is one of the apparent differences between the stimulated and artificial cycles. Similar to a natural cycle with ovulation, the subsequent development of corpus luteum in the stimulated cycle can produce not only steroid hormones but also other products, such as relaxin and vascular endothelial growth factors (VEGFs), which are correlated with the process of decidualization and trophoblast invasion [45–48] and ultimately affect the development of the placenta. Impaired placental development has been demonstrated to play a critical role in preterm birth and preeclampsia [49, 50]. Therefore, the insufficiency of corpus luteum related products can potentially increase the risk of these placenta-related complications. One study has reported that lower relaxin in early pregnancy was related to the increased risk of late-onset preeclampsia [51]. In addition to the effect on placental development, the corpus luteum might also be involved in the adaption of maternal circulation via producing vasoactive hormones. A previous cohort study compared the circulatory adaption via measuring carotid-femoral pulse wave velocity and transit time between pregnant women with and without corpus luteum [52]. They found that the expected decrease of carotid-femoral pulse wave velocity and increased carotid-femoral transit time in normal pregnancy were impaired in women with HRT protocols during the first trimester. These women had a significantly higher incidence of preeclampsia [52]. Therefore, the absence of corpus luteum in artificial FET cycles might be one of the potential causes for the higher risk of preeclampsia. However, the effects on preterm birth have not yet been investigated and current evidence is still very limited. More basic and clinical studies are urgently demanded to investigate the causal link between the absence of corpus luteum and increased risk of pregnancy complications.

### Limitations and further research

There are several limitations to this meta-analysis. As in other meta-analyses, the included studies were heterogeneous in the characteristics of age, embryo fertilization, dosage of estrogen and progesterone administration, stage/number of transferred embryos and definition of pregnancy outcomes. In addition, the effects of pretreatment with GnRH agonist before artificial cycle, the effects of hCG triggering and luteal support after ovulation on the outcomes should also be further addressed in future studies. Furthermore, there was little information on insulin resistance and application of metformin in the included studies. The effects of these confounding factors cannot be further analyzed without individual patient data analysis. Further comparison with more uniform characteristics should be conducted in the future. In addition, there was only 1 RCT study, while the rest 8 studies were all retrospective cohort studies. Therefore, more RCT studies comparing different stimulated protocols are necessary to evaluate which protocol will be optimal for women with PCOS. In addition, there were only 2 studies available for the analysis of obstetric complications between the stimulation cycles and artificial cycle in women with PCOS undergoing IVF-FET. More well-designed studies with larger sample sizes and post-ET following-up are needed to investigate the potential impact of endometrium preparation protocols on the long-term outcomes.

## Conclusions

For endometrial preparation prior to FET in women with PCOS, the stimulation protocol might be superior to HRT protocol with a higher rate of live birth, lower risk of miscarriage, preterm birth and preeclampsia. However, quality of the evidence is low, more well-designed RCT studies are still needed to confirm the results before clinical recommendation, particularly direct comparisons between letrozole and other stimulated regimens.

## Supplementary Information

**Additional file 1.** **Supplementaryfigure 1.** Live birth rate between the stimulated and HRT cycles (without exclusionof study with publication bias). **Supplementaryfigure 2.** Funnel plot for comparison of live birth rate (A) and ongoingpregnancy rate (B). **Supplementaryfigure 3.** Subgroup analyses of different stages of embryo transferred for theoutcome of live birth rate between the stimulated and HRT cycles. **Supplementary figure 4.** Ongoing pregnancy ratebetween the stimulated and HRT cycles (without exclusion of study withpublication bias). **Supplementary figure 5.** Ongoing pregnancy ratebetween the stimulated and HRT cycles (exclusion of study with publicationbias). **Supplementary figure 6.** Clinical pregnancy ratebetween stimulated and HRT cycles. **Supplementary figure 7.** Subgroup analyses for clinicalpregnancy rate between different stimulation drugs and HRT cycles. **Supplementaryfigure 8.** Subgroup analyses of different stages of embryo transferred for theoutcome of miscarriage rate between the stimulated and HRT cycles. **Supplementaryfigure 9.** Implantation rate between the stimulated and HRT cycles. **Supplementaryfigure 10.** Subgroup analyses for implantation rate between different stimulationdrugs and HRT cycles. **Supplementaryfigure 11.** hCG-positive rate between the stimulated and HRT cycles. **Supplementaryfigure 12.** Cycle cancelation rate between the stimulated and HRT cycles. **Supplementary figure 13.** Ectopic pregnancy ratebetween the stimulated and HRT cycles. **Supplementary figure 14.** Analysis of (A)preterm birth rate (per baby); (B) preeclampsia rate, (C) gestationalhypertension rate, (D) gestational diabetes mellitus rate, (E) abnormalplacentation rate (per women with live birth) between the stimulated and HRTcycles. **Supplementary figure 15.** Mechanisms of theaction of letrozole and gonadotropins in ovarian stimulation (Created withBioRender.com).

## Data Availability

The current study was based on results of relevant published studies.

## Abbreviations

PCOS: Polycystic ovary syndrome
OHSS: Ovarian hyperstimulation syndrome
FET: Frozen-embryo transfer
HRT: Hormone replacement therapy
CC: Clomiphene citrate
HMG: Human menopausal gonadotrophin
rFSH: Recombined follicle-stimulating hormone
LH: Luteinizing hormone
RCT: Randomized controlled trials
NOS: Newcastle–Ottawa Scale
LBR: Live birth rate
CPR: Clinical pregnancy rate
MR: Miscarriage rate
IR: Implantation rate
HPR: HCG-positive rate
CCR: Cycle cancellation rate
EPR: Ectopic pregnancy rate
PBR: Preterm birth rate
PR: Preeclampsia rate
GHR: Gestational hypertension rate
GDMR: Gestational diabetes mellitus rate
APR: Abnormal placentation rate
VEGFs: Vascular endothelial growth factors
