# Supplementary material for: Systematic review update and meta-analysis of randomized and non-randomized controlled trials of ovarian stimulation versus artificial cycle for endometrial preparation prior to frozen embryo transfer in women with polycystic ovary syndrome
Source: Reprod Biol Endocrinol. 2022 Apr 2;20:62. doi: 10.1186/s12958-022-00931-4 (PMC8976372; doi:10.1186/s12958-022-00931-4)
Supplement: Supplementary file 1 — Additional file 1. Supplementaryfigure 1. Live birth rate between the stimulated and HRT cycles (without exclusionof study with publication bias). Supplementaryfigure 2. Funnel plot for comparison of live birth rate (A) and ongoingpregnancy rate (B). Supplementaryfigure 3. Subgroup analyses of different stages of embryo transferred for theoutcome of live birth rate between the stimulated and HRT cycles. Supplementary figure 4. Ongoing pregnancy ratebetween the stimulated and HRT cycles (without exclusion of study withpublication bias). Supplementary figure 5. Ongoing pregnancy ratebetween the stimulated and HRT cycles (exclusion of study with publicationbias). Supplementary figure 6. Clinical pregnancy ratebetween stimulated and HRT cycles. Supplementary figure 7. Subgroup analyses for clinicalpregnancy rate between different stimulation drugs and HRT cycles. Supplementaryfigure 8. Subgroup analyses of different stages of embryo transferred for theoutcome of miscarriage rate between the stimulated and HRT cycles. Supplementaryfigure 9. Implantation rate between the stimulated and HRT cycles. Supplementaryfigure 10. Subgroup analyses for implantation rate between different stimulationdrugs and HRT cycles. Supplementaryfigure 11. hCG-positive rate between the stimulated and HRT cycles. Supplementaryfigure 12. Cycle cancelation rate between the stimulated and HRT cycles. Supplementary figure 13. Ectopic pregnancy ratebetween the stimulated and HRT cycles. Supplementary figure 14. Analysis of (A)preterm birth rate (per baby); (B) preeclampsia rate, (C) gestationalhypertension rate, (D) gestational diabetes mellitus rate, (E) abnormalplacentation rate (per women with live birth) between the stimulated and HRTcycles. Supplementary figure 15. Mechanisms of theaction of letrozole and gonadotropins in ovarian stimulation (Created withBioRender.com). [file 12958_2022_931_MOESM1_ESM.docx]

**Supplementary file**

Supplementary figure 1. Live birth rate between the stimulated and HRT cycles (without exclusion of study with publication bias).

Supplementary figure 2. Funnel plot for comparison of live birth rate (A) and ongoing pregnancy rate (B).

Supplementary figure 3. Subgroup analyses of different stages of embryo transferred for the outcome of live birth rate between the stimulated and HRT cycles.

Supplementary figure 4. Ongoing pregnancy rate between the stimulated and HRT cycles (without exclusion of study with publication bias).

Supplementary figure 5. Ongoing pregnancy rate between the stimulated and HRT cycles (exclusion of study with publication bias).

Supplementary figure 6. Clinical pregnancy rate between stimulated and HRT cycles.

Supplementary figure 7. Subgroup analyses for clinical pregnancy rate between different stimulation drugs and HRT cycles.

Supplementary figure 8. Subgroup analyses of different stages of embryo transferred for the outcome of miscarriage rate between the stimulated and HRT cycles.

Supplementary figure 9. Implantation rate between the stimulated and HRT cycles.

Supplementary figure 10. Subgroup analyses for implantation rate between different stimulation drugs and HRT cycles.

Supplementary figure 11. hCG-positive rate between the stimulated and HRT cycles.
Supplementary figure 12. Cycle cancelation rate between the stimulated and HRT cycles.

Supplementary figure 13. Ectopic pregnancy rate between the stimulated and HRT cycles.

Supplementary figure 14. Analysis of (A) preterm birth rate (per baby); (B) preeclampsia rate, (C) gestational hypertension rate, (D) gestational diabetes mellitus rate, (E) abnormal placentation rate (per women with live birth) between the stimulated and HRT cycles.

Supplementary figure 15. Mechanisms of the action of letrozole and gonadotropins in ovarian stimulation (Created with BioRender.com).


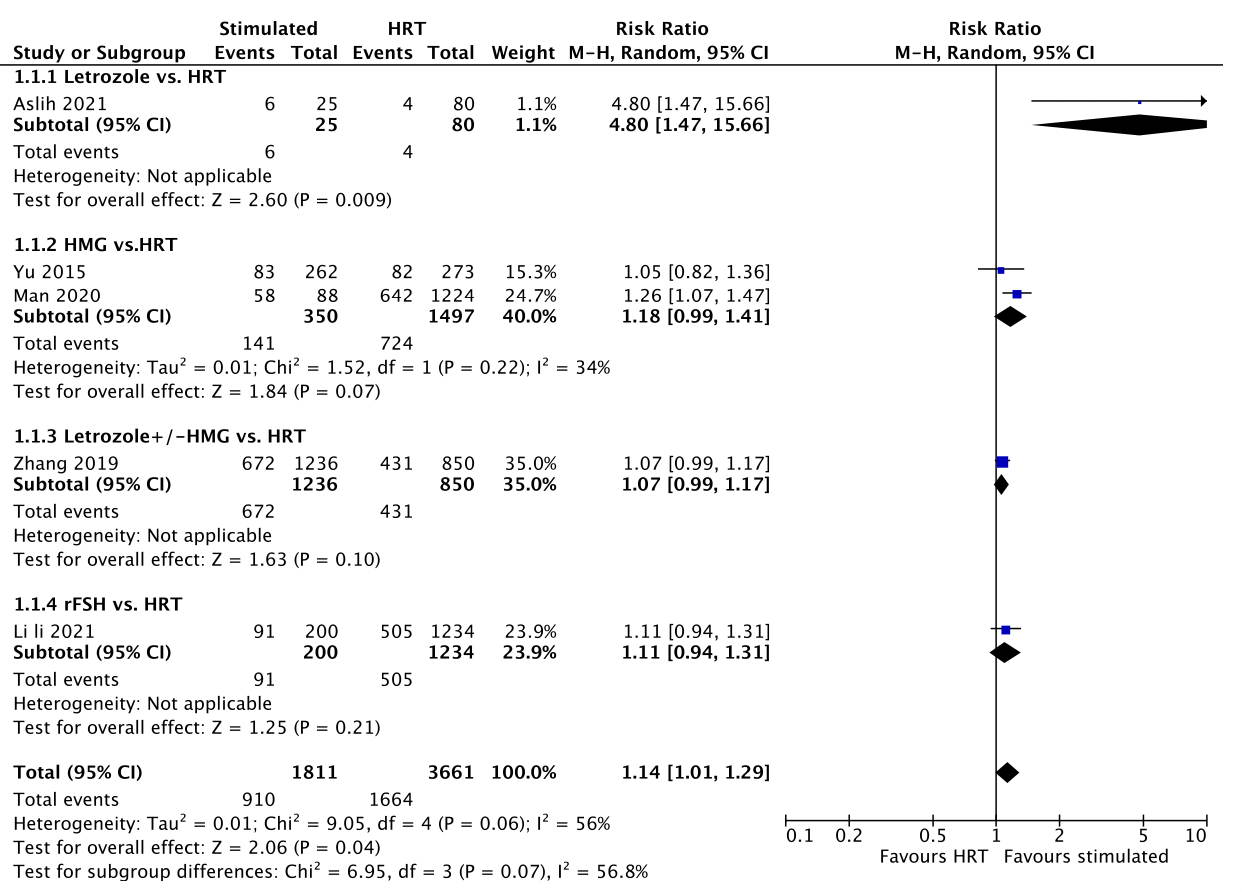
Supplementary figure 1. Live birth rate between the stimulated and HRT cycles (without excluding study with publication bias).


Supplementary figure 2. Funnel plot for comparison of live birth rate (A) and ongoing pregnancy rate (B).


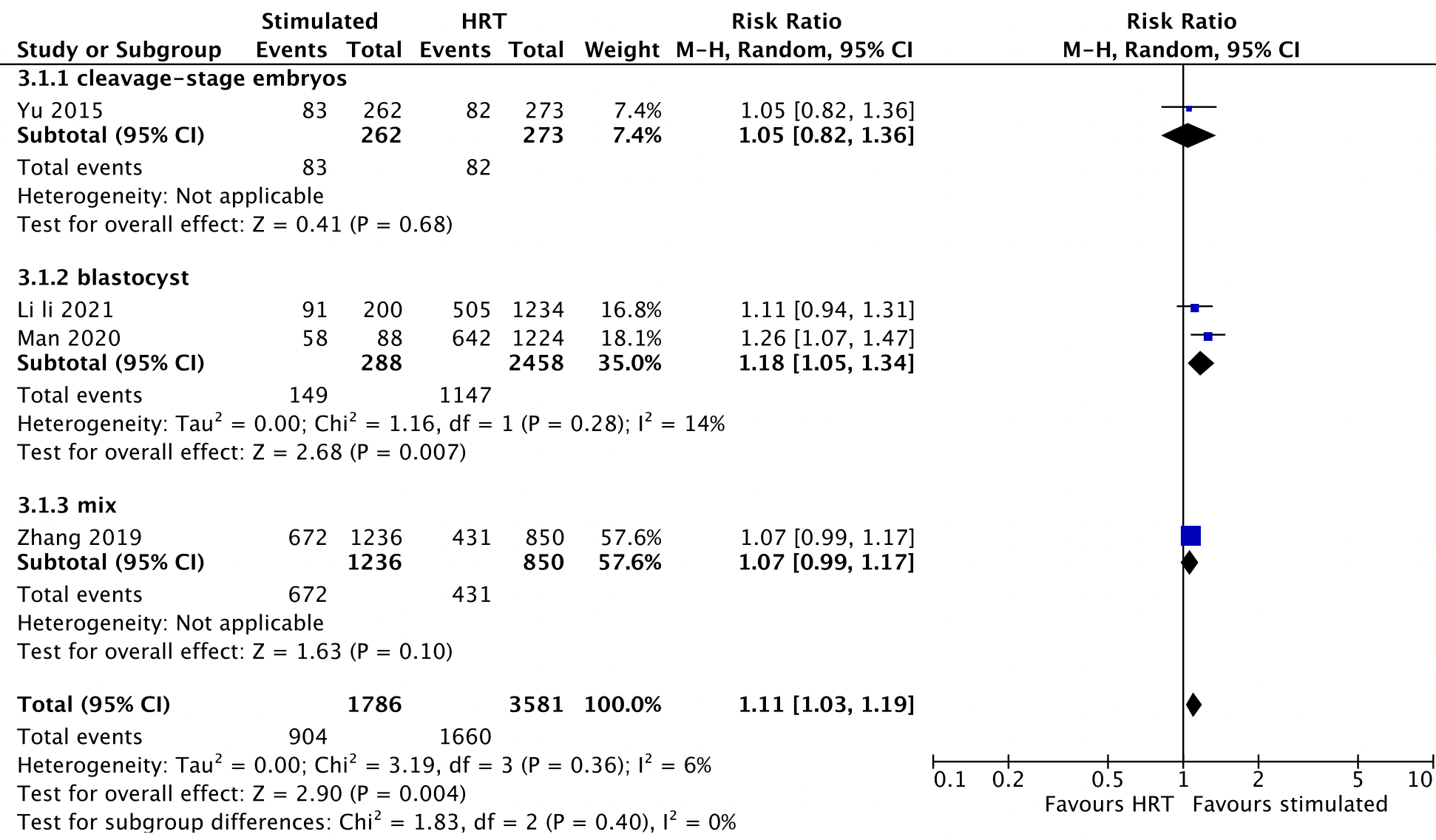

Supplementary figure 3. Subgroup analyses of different stages of embryo transferred for the outcome of live birth rate between the stimulated and HRT cycles.


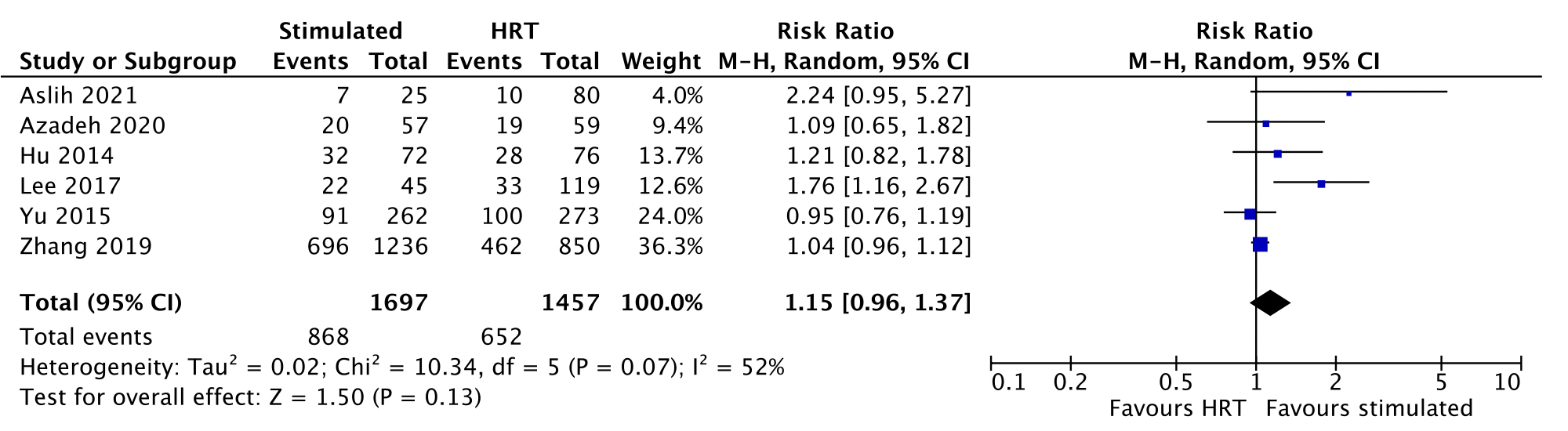


Supplementary figure 4. Ongoing pregnancy rate between the stimulated and HRT cycles (without exclusion of study with publication bias).


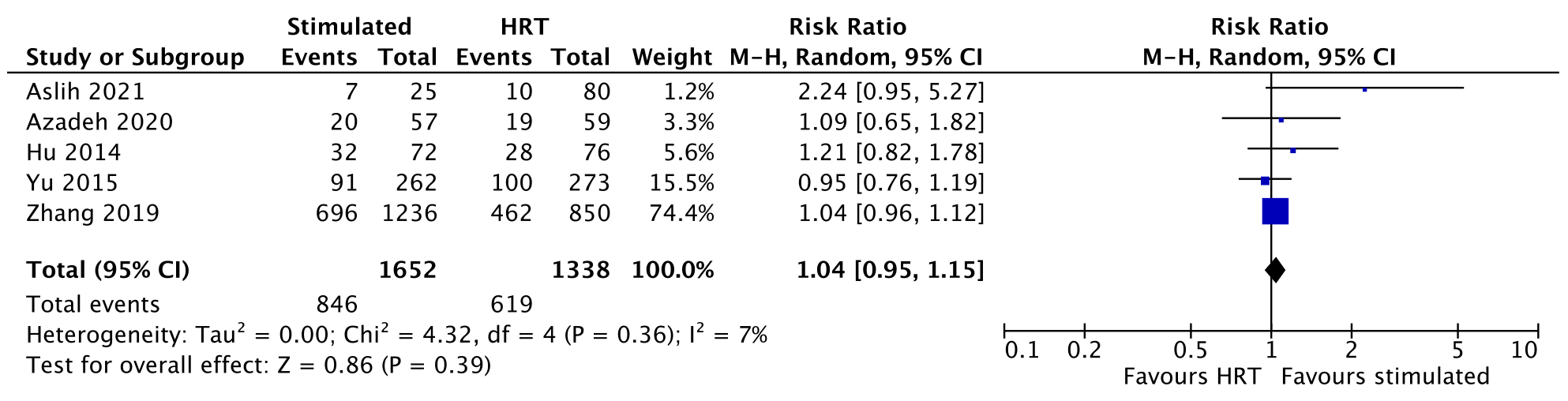

Supplementary figure 5. Ongoing pregnancy rate between the stimulated and HRT cycles (exclusion of study with publication bias).


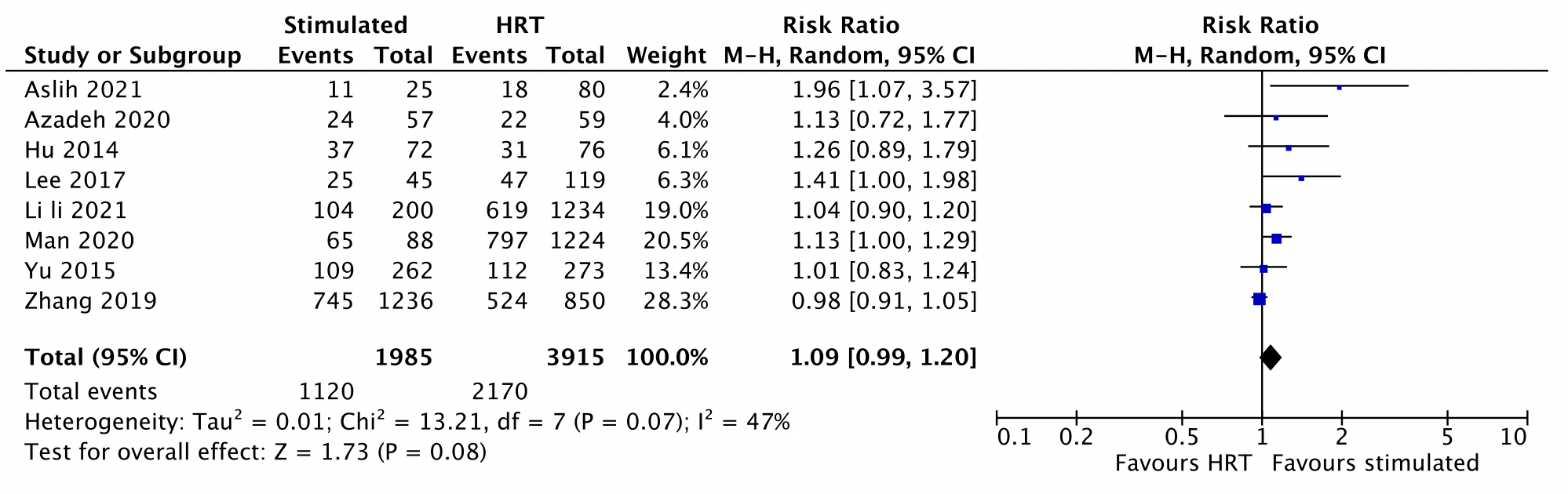
Supplementary figure 6. Clinical pregnancy rate between the stimulated and HRT cycles.


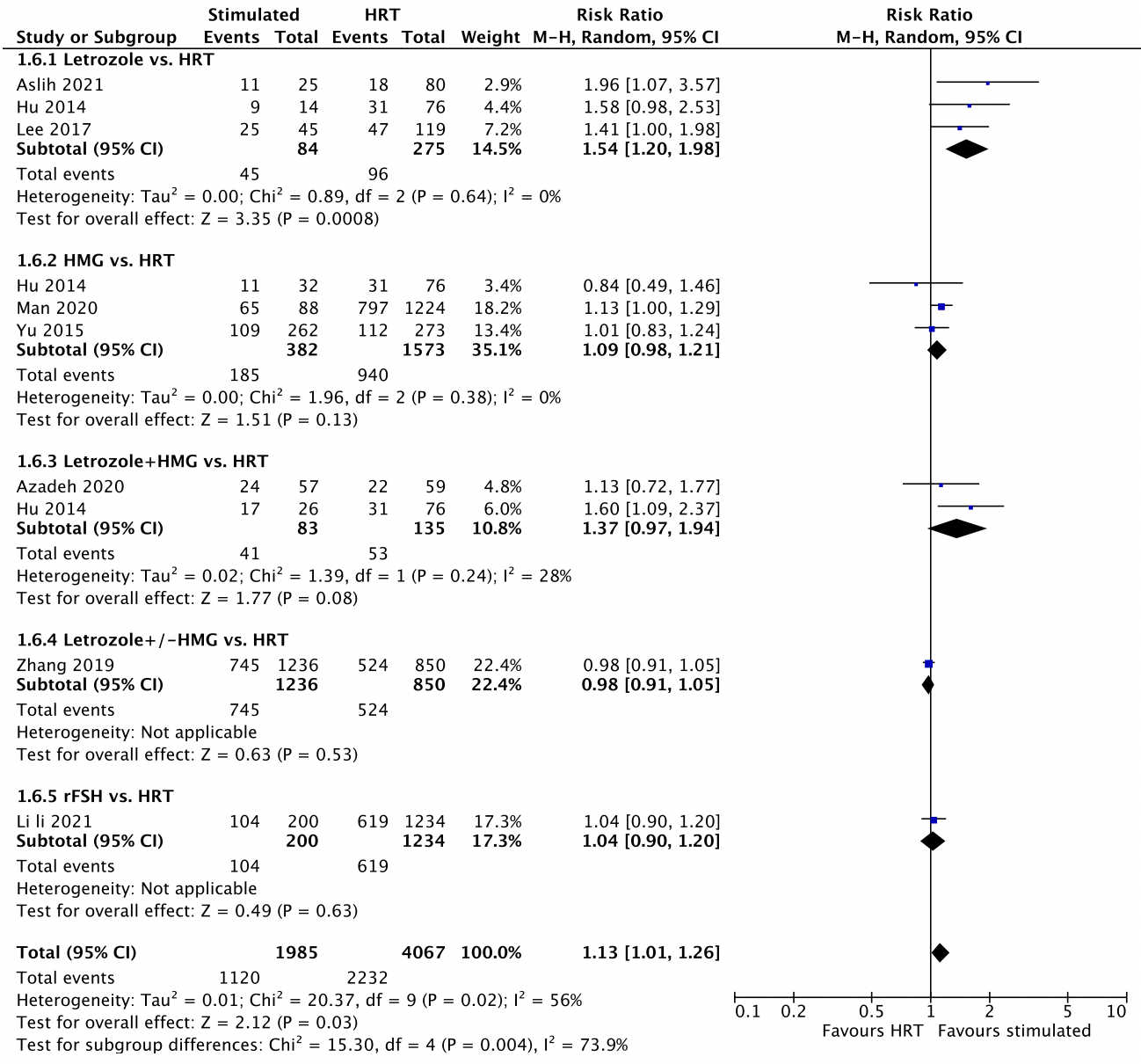

Supplementary figure 7. Subgroup analyses for clinical pregnancy rate between different stimulation drugs and HRT cycles.


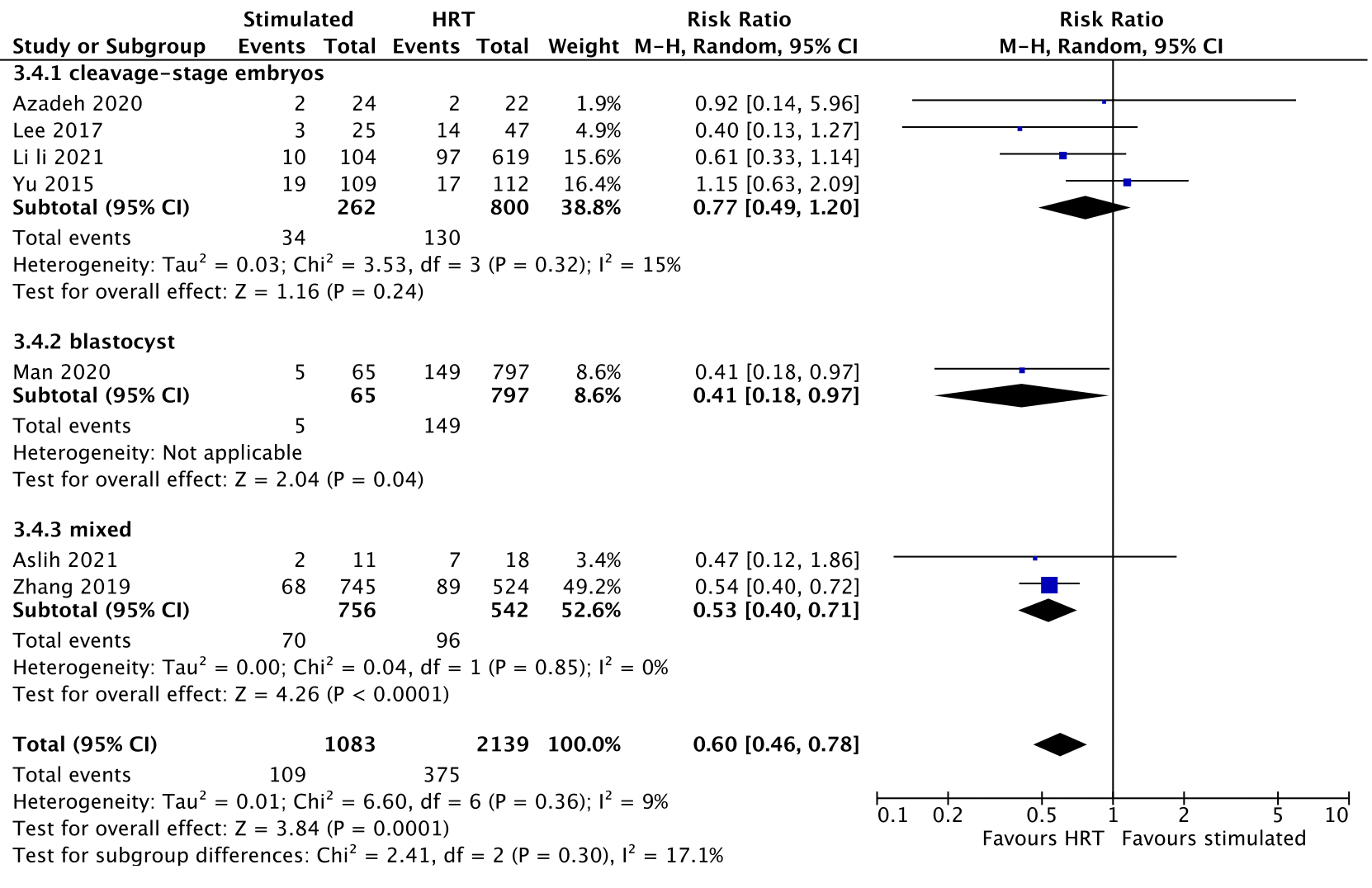

Supplementary figure 8. Subgroup analyses of different stages of embryo transferred for the outcome of miscarriage rate between the stimulated and HRT cycles.

**
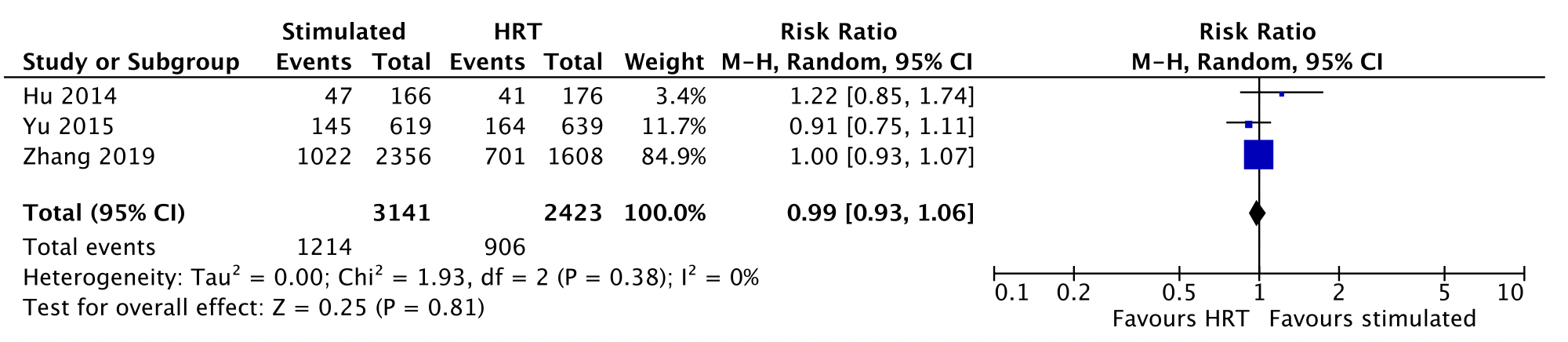
**Supplementary figure 9. Implantation rate between the stimulated and HRT cycles.


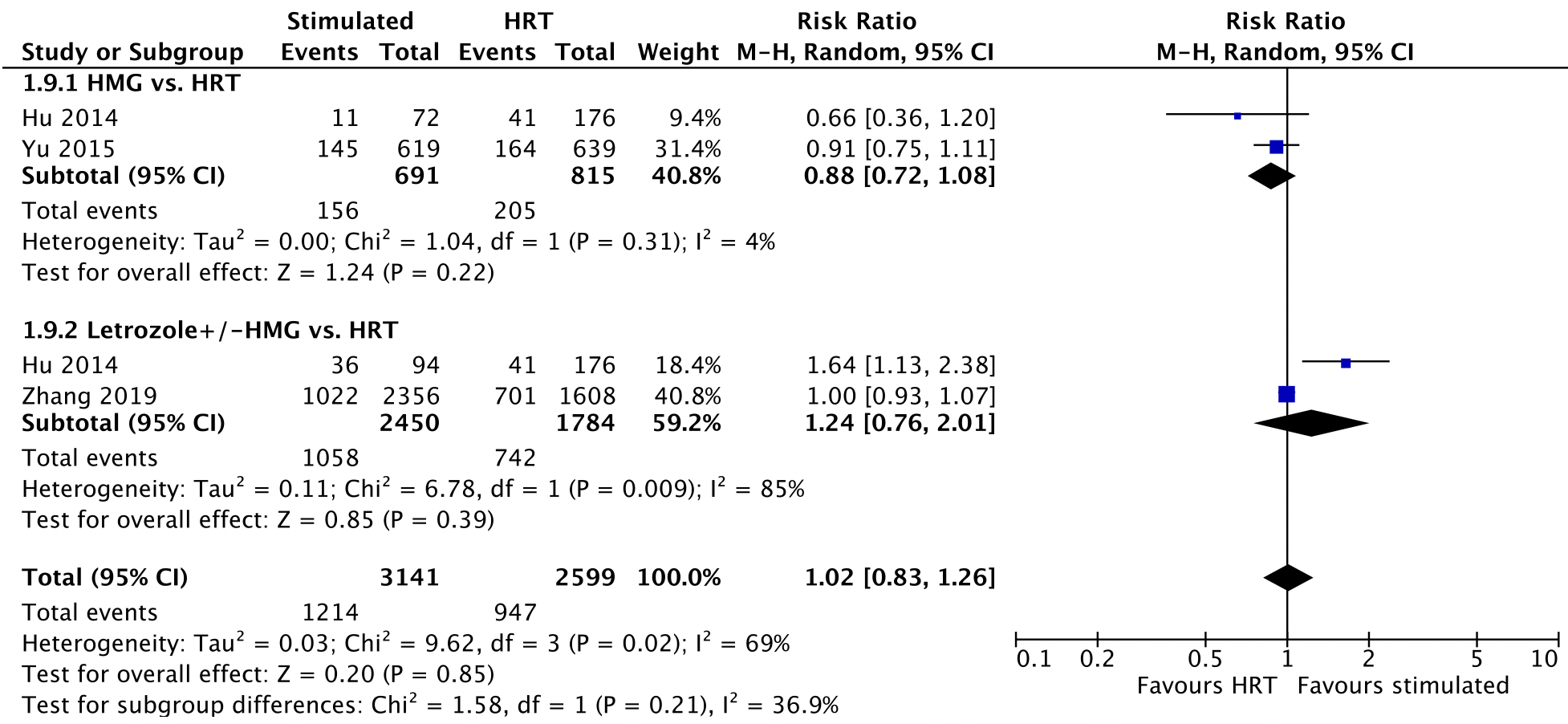

Supplementary figure 10. Subgroup analyses for implantation rate between different stimulation drugs and HRT cycles.


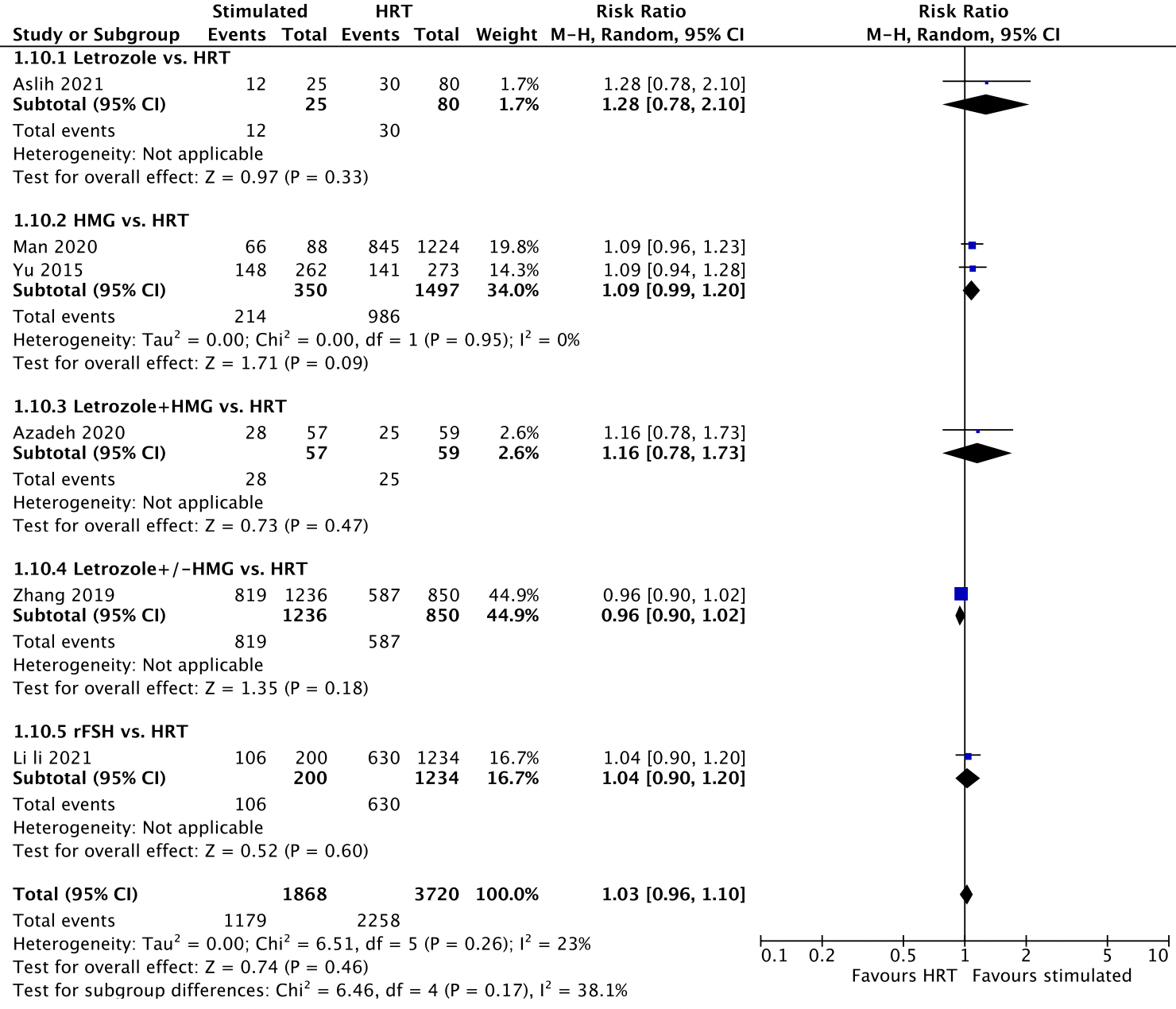
Supplementary figure 11. hCG-positive rate between the stimulated and HRT cycles.


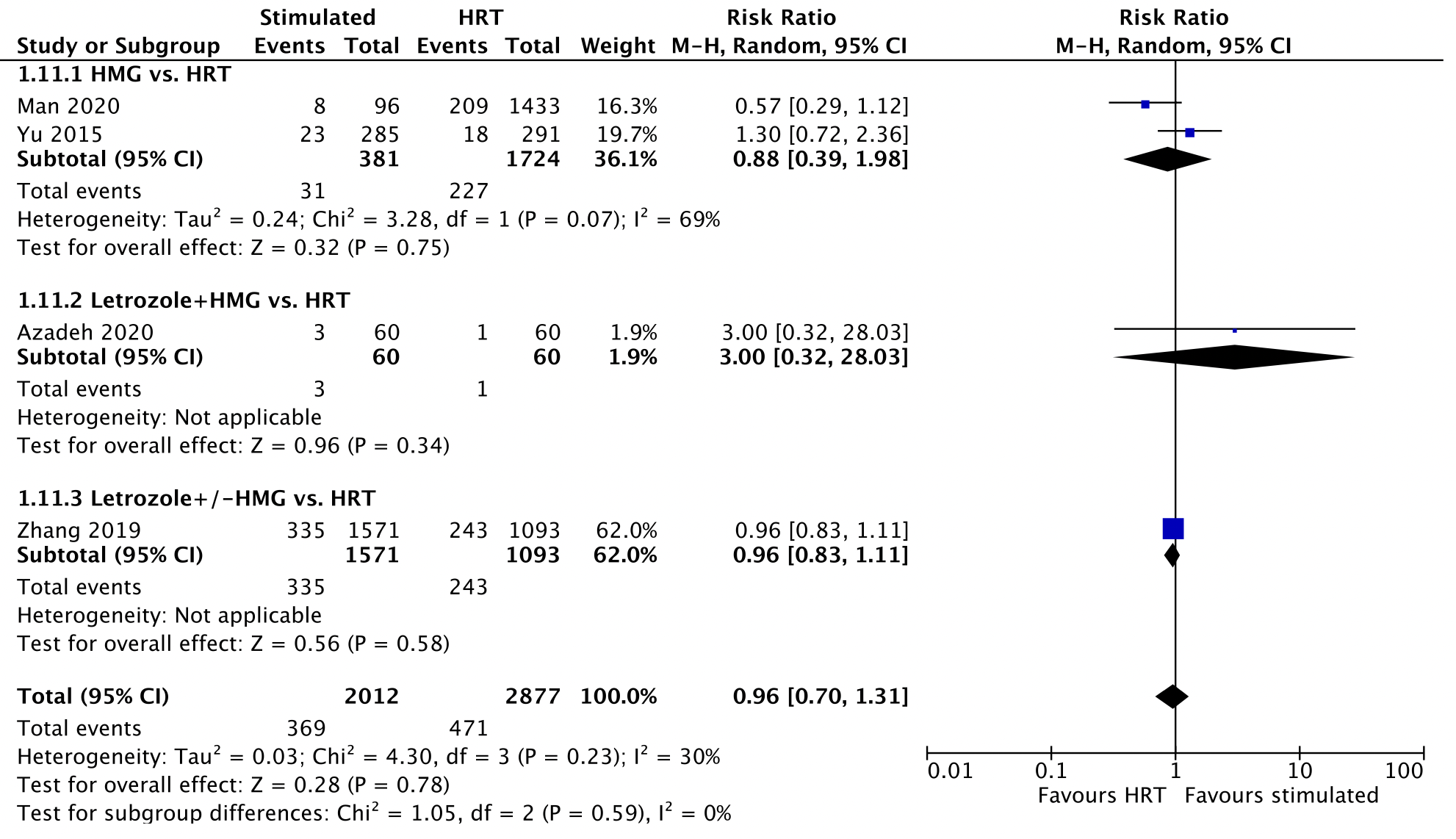

Supplementary figure 12. Cycle cancelation rate between the stimulated and HRT cycles.
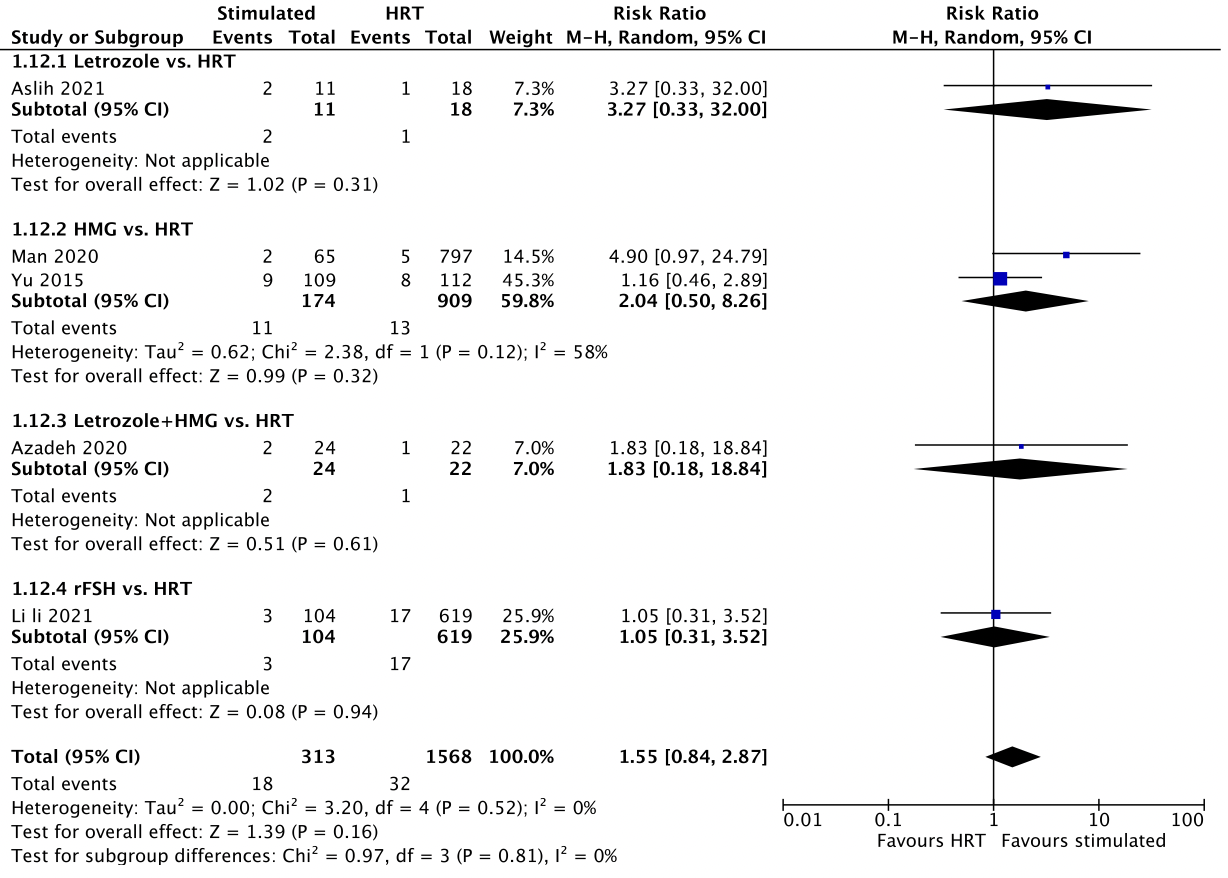
Supplementary figure 13. Ectopic pregnancy rate between the stimulated and HRT cycles.

Supplementary Figure 14. Analysis of (A) preterm birth rate (per baby); (B) preeclampsia rate, (C) gestational hypertension rate, (D) gestational diabetes mellitus rate, (E) abnormal placentation rate (per women with live birth) between the stimulated and HRT cycles.


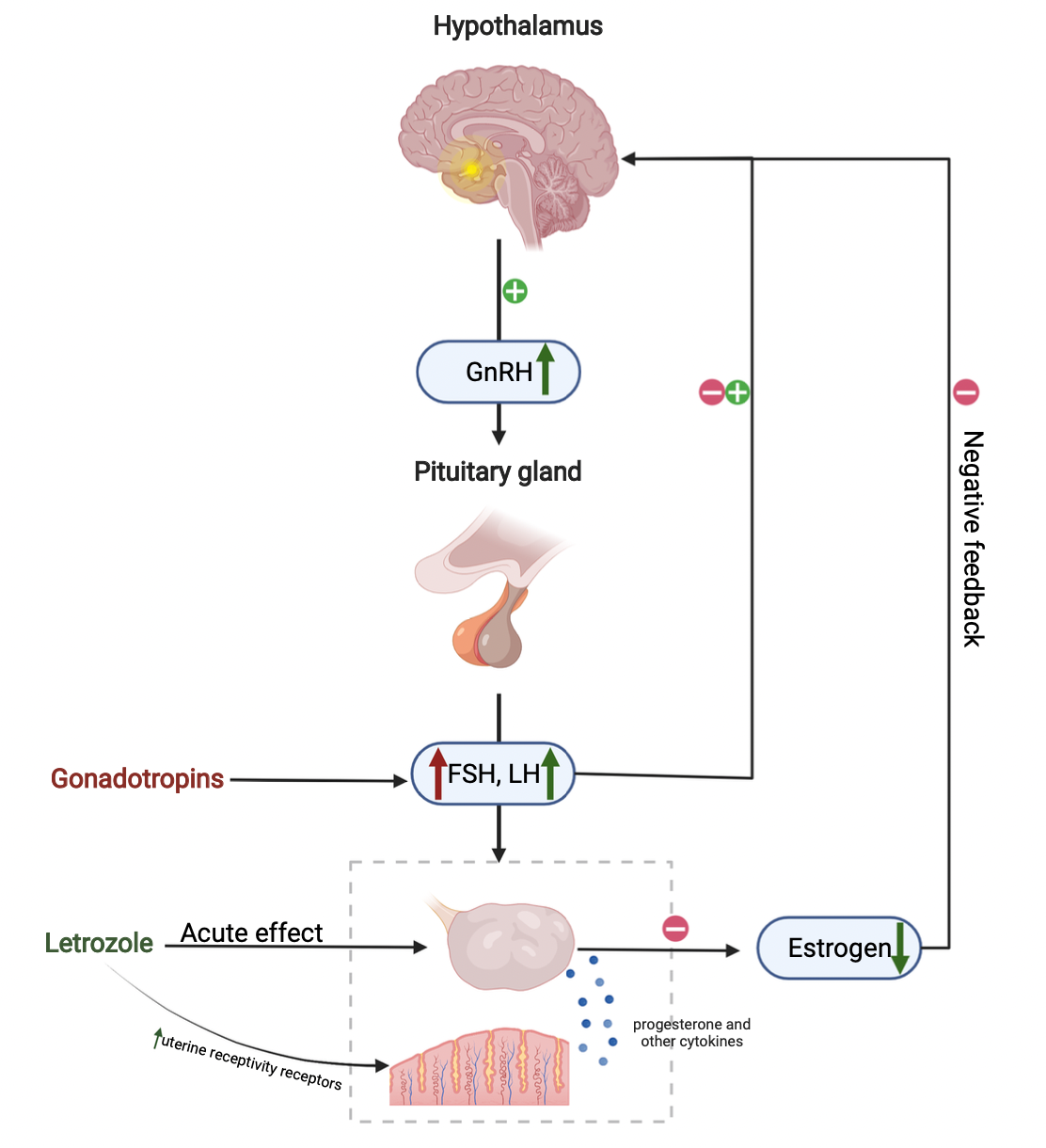

Supplementary figure 15. Mechanisms of the action of letrozole and gonadotropins in ovarian stimulation (Created with BioRender.com).
